# Supplementary material for: Enhanced neutralization potency of an identical HIV neutralizing antibody expressed as different isotypes is achieved through genetically distinct mechanisms
Source: Sci Rep. 2022 Oct 1;12:16473. doi: 10.1038/s41598-022-20141-7 (PMC9526727; doi:10.1038/s41598-022-20141-7)
Supplement: Supplementary file 1 — Supplementary Figure 1. [file 41598_2022_20141_MOESM1_ESM.pdf]

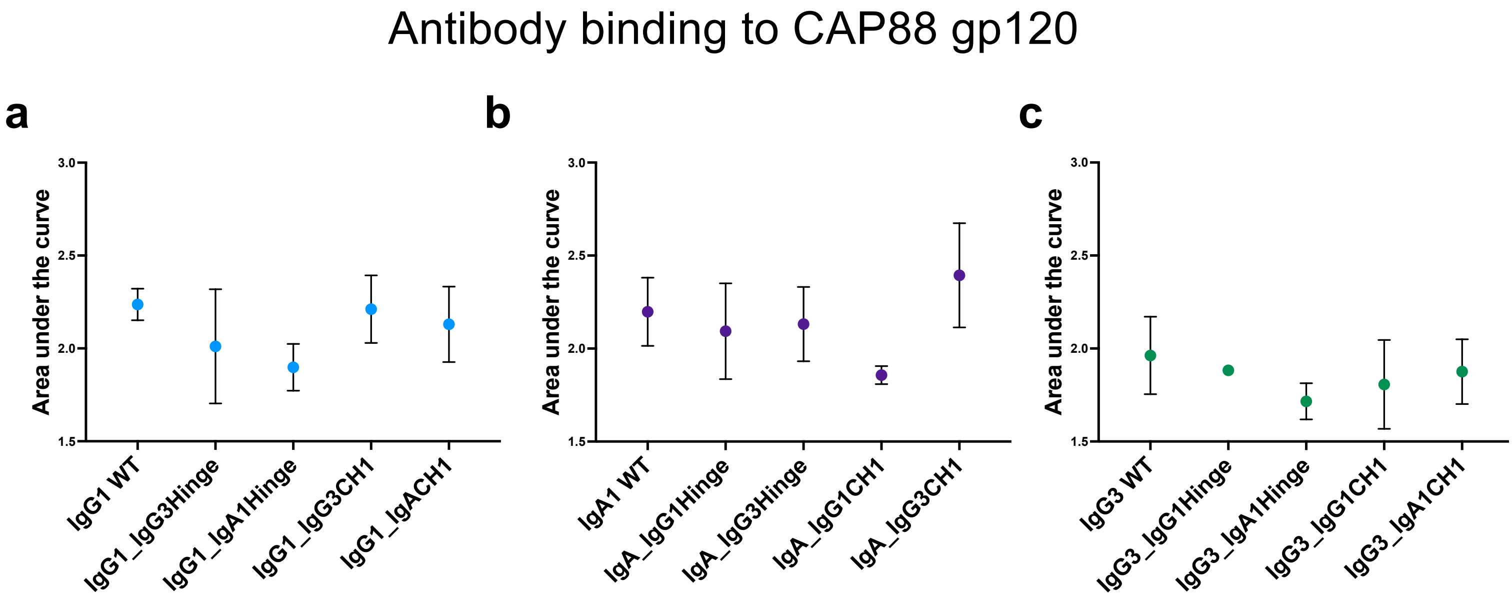

Supplementary Figure 1: CAP88-CH06 antibodies and their hinge and CH1 chimeras bind to the HIV-1 gp120. The CAP88-CH06 (**a**) IgG1, (**b**) IgA1 and (**c**) IgG3 WT antibodies and their respective hinge and CH1 chimeras were tested for binding to an autologous CAP88 gp120 protein using an in-house ELISA. All experiments were conducted in duplicate and error bars represent the mean with standard deviation of three experiments. The area under the curve of each antibody titration curve was calculated and the Krustal-Wallis test with Dunn's correction was performed. No comparisons were significantly different.
